# A Signaling-Threshold Framework for Human Tooth Agenesis: Integrating Molecular Genetics with Developmental Field Theory

**DOI:** 10.3390/ijms27104528

**Published:** 2026-05-18

**Authors:** Anna Ewa Kuc, Paulina Kuc, Natalia Kuc, Magdalena Sulewska, Marzena Tylicka, Michał Sarul

**Affiliations:** 1 Department of Dentofacial Orthopedics and Orthodontics, Wroclaw Medical University, 50-425 Wroclaw, Poland; 2 Faculty of Medicine, Medical University of Bialystok, ul. Kilińskiego 1, 15-089 Bialystok, Poland; 3 Department of Periodontal and Oral Mucosa Diseases, Medical University of Bialystok, ul. Waszyngtona 13, 15-269 Bialystok, Poland; magdalena.sulewska@umb.edu.pl; 4 Department of Medical Biophysics, Medical University of Bialystok, ul. Mickiewicza 2a, 15-089 Bialystok, Poland; marzena.tylicka@umb.edu.pl; 5 Department of Integrated Dentistry, Wroclaw Medical University, 50-425 Wroclaw, Poland; michal.sarul@umw.edu.pl

**Keywords:** hypodontia, oligodontia, odontogenesis, Wnt signaling pathway, bone morphogenetic proteins, gene expression regulation

## Abstract

Tooth agenesis is a common developmental anomaly of the human dentition, ranging from hypodontia to oligodontia, yet its marked phenotypic variability remains insufficiently explained. This review synthesizes developmental and molecular evidence on epithelial–mesenchymal interactions during early odontogenesis and proposes a signaling-threshold framework for human tooth agenesis. We focus on the coordinated roles of Wnt/β-catenin, bone morphogenetic protein (BMP), fibroblast growth factor (FGF), and Sonic hedgehog (SHH) pathways and on recurrent disease-associated genes, including *MSX1*, *PAX9*, *WNT10A*, and *AXIN2*, as quantitative modulators of pathway activity rather than binary determinants of tooth identity. Within this framework, successful tooth initiation may depend on whether integrated signaling output exceeds a field-specific activation threshold within spatially graded developmental regions of the dental arch. Differences in signaling amplitude, duration, and transcriptional responsiveness may therefore account for distal tooth susceptibility, variable penetrance, arch asymmetry, and the broad clinical spectrum from mild hypodontia to severe oligodontia. By integrating molecular genetics with developmental field theory, this model provides a testable systems-level explanation for selective tooth absence and highlights priority directions for future functional and genotype–phenotype studies.

## 1. Introduction

Tooth agenesis represents one of the most common developmental anomalies of the craniofacial complex, encompassing a spectrum from mild hypodontia to severe oligodontia. Although numerous genetic variants have been identified in affected individuals, the mechanisms by which similar mutations produce highly variable phenotypes remain insufficiently explained. Clinical observations demonstrate striking heterogeneity: some individuals exhibit absence of a single tooth, whereas others lack multiple posterior teeth despite carrying variants within the same signaling pathways. This variability suggests that tooth initiation is influenced not only by linear gene–phenotype relationships but also by regulatory dynamics within odontogenic signaling networks.

Tooth development depends on reciprocal interactions between oral epithelium and neural crest–derived mesenchyme, coordinated through tightly regulated molecular pathways during the bud and cap stages of morphogenesis [1,2]. Central regulatory networks include Wnt/β-catenin, bone morphogenetic protein (BMP), fibroblast growth factor (FGF), and Sonic hedgehog (SHH) signaling [3,4,5,6]. Disruption of these pathways in animal models results in arrest of tooth development at early morphogenetic stages, confirming their essential role in odontogenesis [3,5,7].

In humans, pathogenic variants in genes such as *MSX1*, *PAX9*, *WNT10A*, and *AXIN2* have been consistently associated with non-syndromic hypodontia and oligodontia [8,9,10,11]. These genes function as modulators of signaling intensity or transcriptional responsiveness within the odontogenic network rather than as isolated determinants of individual tooth identity. Notably, the distribution of missing teeth is non-random but varies according to population, dentition type, and whether third molars are included. When third molars are excluded, mandibular second premolars, maxillary lateral incisors, and maxillary second premolars are repeatedly reported among the most commonly absent permanent teeth, supporting the view that specific odontogenic fields differ in susceptibility to developmental perturbation [12,13,14,15,16].

These non-random genotype–phenotype patterns make two concepts particularly relevant for the present review. Here, a developmental field refers to a spatially organized odontogenic region in which neighboring tooth germs share timing, tissue competence, and signaling inputs, whereas the term threshold denotes the minimal integrated signaling activity required for successful initiation of a tooth germ. Together, these concepts connect classical developmental theory with current molecular evidence [12,13].

Despite these advances, current literature still tends to describe associations between specific genes and missing teeth without integrating molecular signaling, spatial field gradients, and phenotypic variability into a unified framework. As a result, a biologically coherent explanation for differential tooth absence, arch-specific patterns, and variable penetrance remains incomplete.

Recent reviews have summarized developmental patterns of tooth-number regulation and catalogued the expanding genetic landscape of tooth agenesis [17,18]. However, they have more often emphasized either descriptive genotype–phenotype associations or field-based patterning than the quantitative behavior of integrated signaling networks. The present review therefore asks whether variation in pathway dosage and epithelial–mesenchymal crosstalk may help explain why similar genetic perturbations produce different patterns and severities of agenesis.

The aim of this review is to synthesize current developmental, molecular, and clinical evidence and to propose a signaling-threshold framework for human tooth agenesis. In this framework, pathway activity is interpreted as a quantitative, field-dependent determinant of tooth initiation rather than as a simple one-gene/one-tooth relationship. The distinctive feature of the proposed model, relative to earlier threshold-based and developmental field concepts, is the explicit integration of molecular pathway dosage, epithelial–mesenchymal signaling crosstalk, and field-specific susceptibility into a single testable systems-level interpretation.

The threshold concept also has important historical roots in dental developmental biology. Brook proposed a multifactorial model in which anomalies of tooth number and size are distributed along a continuous scale, with thresholds separating normal variation from hypodontia, hyperdontia, microdontia, and macrodontia [19]. The present review does not replace this classical view; rather, it builds on it by linking threshold behavior to specific molecular processes. In particular, the proposed framework adds three mechanistic components that were not explicit in earlier models: pathway dosage within Wnt/BMP/FGF/SHH signaling networks, epithelial–mesenchymal crosstalk as the integrative substrate of initiation failure, and field-specific molecular susceptibility across the dental arch. In this sense, the framework is intended as a molecular and spatial refinement of earlier multifactorial threshold concepts.

**Figure 1:**
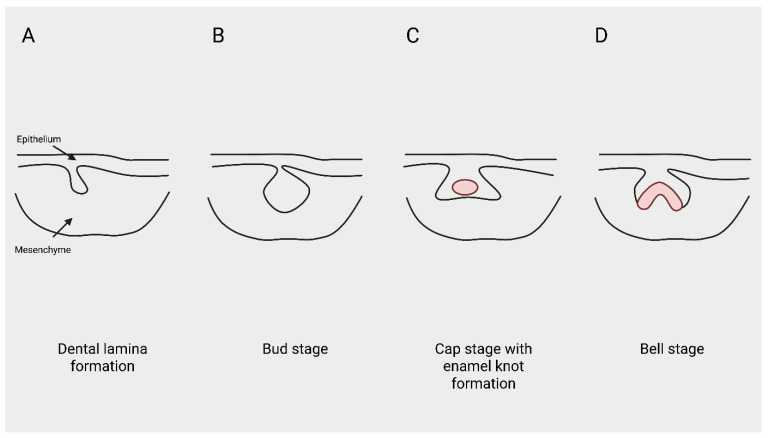
Schematic representation of the sequential stages of early human tooth development. (**A**) Formation of the dental lamina through epithelial thickening. (**B**) Bud stage characterized by epithelial invagination into neural crest–derived mesenchyme. (**C**) Cap stage with enamel knot formation as a transient epithelial signaling center. (**D**) Bell stage illustrating advanced morphogenetic organization. Created in BioRender. Anna Ewa Kuc (2026).

**Figure 2:**
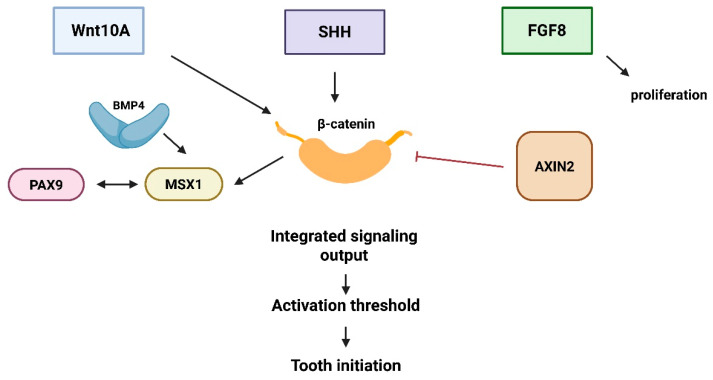
Schematic representation of the integrated odontogenic signaling network underlying tooth initiation. The diagram summarizes reciprocal epithelial–mesenchymal interactions among Wnt/β-catenin, BMP, FGF, and SHH pathways and illustrates how integrated pathway activity contributes to the activation threshold required for successful tooth initiation. Created in BioRender. Anna Ewa Kuc (2026).

**Figure 3:**
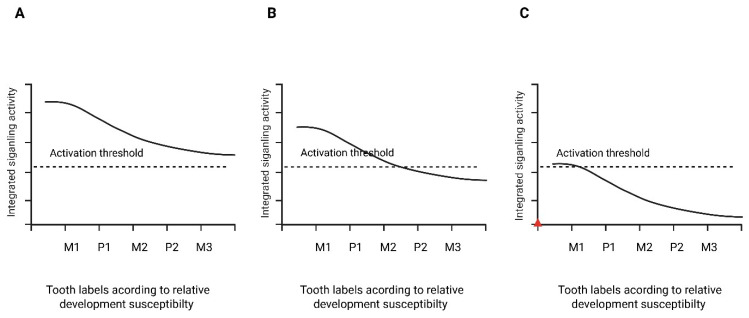
Conceptual signaling-threshold model of tooth initiation. The figure is schematic and not data-derived. Integrated signaling activity decreases along a developmental susceptibility gradient. The dashed line represents the activation threshold required for tooth initiation. (**A**) Normal signaling exceeds threshold across all fields. (**B**) Mild reduction results in failure of initiation in more susceptible fields. (**C**) Severe reduction produces broader agenesis across the gradient. Tooth labels are arranged according to relative developmental susceptibility rather than strict anatomical sequence. Created in BioRender. Anna Ewa Kuc (2026).

The main developmental stages, signaling interactions, and the proposed threshold concept are summarized schematically in Figure 1, Figure 2 and Figure 3.

### Search Strategy and Selection Rationale

This narrative review was designed to integrate developmental, molecular, and clinical evidence relevant to human tooth agenesis. PubMed, Scopus, and Google Scholar were searched for articles published up to February 2026 using combinations of the following terms: “tooth agenesis”, “hypodontia”, “oligodontia”, “odontogenesis”, “tooth initiation”, “developmental field”, “threshold model”, “Wnt”, “BMP”, “FGF”, “SHH”, “*MSX1*”, “*PAX9*”, “*WNT10A*”, “*AXIN2*”, “*EDA*”, “*BMPR2*”, “*KCTD1*”, and “*FGF8*”. Representative search strings included “tooth agenesis AND *WNT10A*”, “tooth agenesis AND threshold”, “hypodontia AND developmental field”, “tooth development AND single-cell”, and “*AXIN2* AND tooth agenesis AND colorectal cancer”.

Studies were prioritized according to three principles: conceptual relevance to the proposed framework, recency of evidence, and direct relevance to human tooth agenesis. Preference was given to original human genetic studies, genotype–phenotype analyses, and recent reviews when these were available. Animal and in vitro studies were included primarily to support mechanistic interpretation of epithelial–mesenchymal signaling, pathway crosstalk, and developmental timing when such processes cannot be directly examined during early human odontogenesis. Articles were excluded when they focused exclusively on treatment mechanics, restorative management, eruption disorders, or dental anomalies unrelated to tooth initiation. Additional records were identified through backward citation tracking of key reviews and primary studies. Because the aim of this article was conceptual integration rather than quantitative evidence synthesis, the review was conducted as a narrative review and not as a systematic review or meta-analysis; therefore, no PRISMA flow diagram was generated.

## 2. Tooth Development

Tooth development is initiated through reciprocal signaling interactions between the oral epithelium and neural crest–derived mesenchyme, beginning with localized thickening of the dental lamina along the embryonic jaw arches [1,3]. Canonical Wnt signaling within the epithelium is essential for establishing odontogenic competence and activating mesenchymal responsiveness required for bud formation [3,7]. Experimental suppression of early Wnt activity results in failure of tooth initiation rather than structural malformation, underscoring its role as a primary activation signal in odontogenesis [4,7].

During the bud stage, epithelial invagination into the underlying mesenchyme is reinforced by FGF-mediated proliferation and mesenchymal transcriptional regulation involving *MSX1* and *PAX9* [5,6]. These transcription factors integrate upstream signaling inputs and stabilize odontogenic identity. Genetic disruption of *MSX1* or *PAX9* leads to arrest prior to or during bud formation, demonstrating that early signaling intensity and mesenchymal competence are tightly coupled [5,6,9].

Progression to the cap stage introduces the enamel knot, a transient epithelial signaling center that orchestrates spatial patterning through expression of SHH, BMPs, FGFs, and Wnt modulators [20,21]. The enamel knot regulates localized cell proliferation and apoptosis, thereby defining cusp patterning and crown architecture. Importantly, enamel knot formation depends on sustained upstream signaling activity, indicating that morphogenetic organization is contingent upon successful initiation [20,21,22].

Transition to the bell stage involves differentiation of ameloblasts and odontoblasts under continued influence of BMP and FGF gradients [1,23]. However, by this stage, successful initiation has already occurred. Experimental attenuation of Wnt, BMP, or FGF activity during early morphogenesis consistently produces absence of tooth germs rather than malformed but present teeth, highlighting initiation as the principal vulnerability point in odontogenesis [4,5,7]—Figure 1.

Spatial organization of developing teeth along the dental arch reflects graded signaling environments rather than discrete identity switches. Developmental field theory proposes that tooth classes emerge within overlapping morphogenetic fields characterized by differential signaling strength and positional sensitivity [2,12]. Posterior tooth germs arise in regions of comparatively reduced signaling redundancy and are therefore more susceptible to perturbations in pathway activity. This gradient-dependent vulnerability is reflected clinically by the disproportionate absence of second premolars and third molars in hypodontia [12].

Recent single-cell profiling of human tooth development provides an important human-specific context for these mechanistic models. Human fetal odontogenic tissues contain distinct epithelial and mesenchymal cell populations with stage-specific transcriptional programs, demonstrating that human odontogenesis is organized through coordinated cellular differentiation rather than uniform tissue-level signaling alone [24]. Although such datasets do not yet define numerical thresholds for tooth initiation, they provide a framework for identifying the epithelial and mesenchymal subpopulations in which pathway dosage and transcriptional responsiveness could be measured in future studies.

Collectively, normal tooth initiation depends on the integrated magnitude, timing, and spatial distribution of signaling activity within epithelial–mesenchymal networks. Odontogenesis therefore represents a dynamic activation process in which cumulative pathway activity must reach sufficient intensity to trigger and sustain tooth germ formation within each developmental field.

## 3. Signaling Networks

Tooth initiation and early morphogenesis are regulated by an integrated network of signaling pathways rather than by isolated gene effects (Figure 2). Among these, canonical Wnt/β-catenin signaling functions as a central activator of odontogenic competence within the dental epithelium and underlying mesenchyme [3,7]. Stabilization of β-catenin promotes epithelial proliferation and mesenchymal transcriptional activation, whereas disruption of pathway components leads to failure of tooth bud formation [4,7]. Experimental models demonstrate that sustained Wnt activity is required not only for initiation but also for maintenance of the odontogenic field during early morphogenesis [4,25].

Canonical Wnt/β-catenin signaling functions as a central regulatory hub integrating epithelial inputs *(WNT10A*, *SHH*, *FGF8)* with mesenchymal transcriptional modulators *(MSX1* and *PAX9).* BMP4 enhances mesenchymal transcriptional responsiveness, while *AXIN2* acts as a negative regulator of β-catenin stabilization. *FGF8* contributes to proliferative expansion during early morphogenesis. Integrated pathway activity determines cumulative signaling output relative to the developmental activation threshold required for successful tooth initiation.

BMP signaling operates in close coordination with Wnt pathways. BMP4, expressed in both epithelium and mesenchyme, regulates differentiation and spatial patterning, partly through modulation of *MSX1* expression [5,26]. Functional interactions between Wnt and BMP signaling establish positive feedback loops that stabilize early tooth germ formation [5]. Attenuation of BMP signaling reduces mesenchymal responsiveness and may prevent transition from lamina to bud stage, further supporting the view that cumulative signaling intensity determines successful initiation [5,26].

FGF signaling contributes primarily to proliferative expansion and epithelial–mesenchymal communication during bud and cap stages [20,21,22]. *FGF8* expression in the early dental epithelium establishes positional information along the jaw, influencing tooth identity and field boundaries [27]. Experimental perturbation of FGF gradients alters spatial organization of tooth germs, indicating that quantitative variation in FGF signaling affects both initiation probability and patterning outcomes [27,28].

SHH signaling, expressed prominently in the enamel knot, refines morphogenetic organization and regulates proliferation–apoptosis balance during cusp formation [20,21]. Although SHH primarily influences morphogenesis after initiation, its expression depends on upstream Wnt and BMP activity, reinforcing the hierarchical structure of odontogenic regulation [20]. Thus, downstream patterning events remain contingent upon sufficient early pathway activation.

Importantly, these pathways do not operate independently. Cross-regulatory interactions between Wnt, BMP, FGF, and SHH signaling create a nonlinear regulatory network in which pathway intensity, timing, and spatial distribution collectively determine developmental outcomes [4,21]. For example, *AXIN2* functions as a negative regulator within the Wnt pathway, modulating β-catenin stability and thereby influencing the amplitude of signaling responses [10]. Variants affecting *AXIN2* alter pathway dynamics rather than eliminating signaling entirely, illustrating how quantitative modulation rather than complete loss of function may underlie variable phenotypes.

The integration of these signaling systems suggests that tooth initiation emerges from cumulative pathway activity rather than binary on–off genetic switches. Developmental robustness is maintained through redundancy and feedback; however, reduction in signaling amplitude or duration may shift the system toward a critical boundary below which odontogenic programs fail to activate. Such network-level properties provide a mechanistic basis for understanding how modest genetic variation can yield substantial differences in tooth number and distribution.

A key property of this network is developmental robustness. During normal odontogenesis, reciprocal epithelial–mesenchymal signaling and feedback loops between Wnt and BMP pathways stabilize odontogenic competence and buffer minor fluctuations in morphogen availability [4,5,20,26]. This buffering capacity helps explain why partial reduction in pathway activity does not uniformly cause agenesis in every carrier of a pathogenic variant. Instead, the phenotypic outcome depends on how far signaling output is shifted relative to the local activation threshold and whether compensatory inputs from parallel pathways remain sufficient to sustain initiation. From this perspective, variable penetrance is not an exception to gene-based models but an expected feature of a nonlinear signaling system.

Temporal context is equally important. The biological consequences of perturbing Wnt, BMP, FGF, or SHH signaling depend not only on pathway amplitude but also on the developmental stage at which disruption occurs [20,21,22,27,28]. Early reductions in epithelial Wnt activity or mesenchymal BMP responsiveness primarily compromise competence and bud initiation, whereas later perturbations more often influence cusp patterning, crown size, or morphogenetic refinement. This stage dependence reinforces the central argument of the present review: agenesis is best understood as failure of an early activation program, while many later dental anomalies reflect modulation of morphogenesis after initiation has already been secured.

## 4. Genetic Modulation

Genetic variants associated with tooth agenesis primarily affect regulators of odontogenic signaling rather than structural components of the developing tooth germ. Among the most consistently implicated genes are *MSX1*, *PAX9*, *WNT10A*, and *AXIN2*, all of which participate directly or indirectly in modulation of Wnt and BMP pathway activity [6,9,11,29,30]. These genes do not encode tooth-specific determinants; instead, they regulate transcriptional responsiveness and signaling intensity within epithelial–mesenchymal networks that govern initiation.

*PAX9* encodes a paired box transcription factor essential for mesenchymal competence during early odontogenesis. Recent genotype–phenotype analyses of *PAX9*-related nonsyndromic tooth agenesis further support a strong association between *PAX9* variation and posterior tooth susceptibility [31]. Heterozygous mutations are frequently associated with oligodontia, particularly affecting posterior teeth [9,29]. Experimental models demonstrate that reduced *PAX9* expression diminishes BMP-mediated transcriptional activation, compromising stabilization of the tooth bud [5,29]. Importantly, *PAX9* mutations often exhibit incomplete penetrance and variable expressivity, suggesting that reduced transcriptional output may shift signaling activity toward a critical boundary rather than abolish it entirely.

Recent evidence further supports the relevance of BMP pathway dosage in nonsyndromic oligodontia. Variants in *BMPR2* have been implicated in nonsyndromic oligodontia, extending the genetic landscape beyond transcription factors such as *MSX1* and *PAX9* toward receptor-level modulation of BMP responsiveness [32]. This observation is consistent with a threshold model in which impaired ligand–receptor signaling may reduce mesenchymal competence sufficiently to compromise initiation in susceptible tooth fields.

Human genotype–phenotype studies further support the idea that the pattern of missing teeth carries biological information. A systematic analysis of dental agenesis phenotypes linked to causative genes showed that agenesis patterns may help guide molecular diagnosis, indicating that tooth distribution is not merely a descriptive clinical feature but a potential readout of disrupted developmental programs [33]. This evidence strengthens the threshold framework by suggesting that different genes may shift shared signaling systems in ways that preferentially affect particular odontogenic fields.

Similarly, *MSX1* functions as a downstream mediator of BMP signaling and is required for progression beyond the bud stage [6,30]. Loss-of-function variants in *MSX1* are associated with hypodontia affecting premolars and third molars. Experimental evidence indicates that *MSX1* deficiency lowers mesenchymal responsiveness to BMP signals, impairing reinforcement of epithelial–mesenchymal feedback loops necessary for sustained initiation [5,6]. These observations support a model in which *MSX1* modulates signaling amplitude rather than acting as an isolated switch for specific tooth types.

Variants in *WNT10A* represent one of the most common genetic findings in non-syndromic hypodontia [11]. As a ligand within the canonical Wnt pathway, *WNT10A* influences β-catenin stabilization and downstream transcriptional activation. Reduced *WNT10A* activity attenuates epithelial signaling strength and compromises mesenchymal activation, frequently resulting in absence of second premolars and third molars [11]. The broad phenotypic range observed among carriers—from single missing teeth to severe oligodontia—further supports the concept that quantitative modulation of signaling intensity determines clinical outcome.

*AXIN2*, a negative regulator of Wnt signaling, provides additional insight into pathway-level modulation [10]. Variants in *AXIN2* alter β-catenin degradation dynamics and thus influence pathway amplitude rather than eliminating signaling entirely. Individuals carrying *AXIN2* mutations exhibit hypodontia with marked variability in tooth number and distribution, reinforcing the principle that network-level signaling thresholds may underlie phenotypic heterogeneity [10].

Beyond canonical Wnt- and BMP-centered regulators, the *EDA–EDAR–EDARADD*/NF-κB axis should also be considered within a threshold framework of tooth initiation. *EDA* signaling plays a central role in ectodermal organ development and contributes to tooth number regulation in both syndromic and non-syndromic contexts. Recent human evidence suggests that *EDA* dosage may be particularly important in deciduous dentition, while broader pathway interactions indicate that *EDA* signaling can modify the overall responsiveness of odontogenic tissues rather than determine a single tooth fate in isolation. Incorporating *EDA* into the present framework strengthens the interpretation of tooth agenesis as a network-level consequence of altered signaling amplitude across developmentally sensitive fields [34].

Recent studies also broaden the set of pathway modulators relevant to tooth agenesis. *KCTD1* variants have been associated with isolated dental anomalies and shown to affect β-catenin levels, supporting a role for WNT–SHH–BMP pathway crosstalk in variable dental phenotypes. Experimental dysregulation of mesenchymal *FGF8* signaling has likewise been shown to produce incisor agenesis and molar microdontia, underscoring how quantitative disturbance of pathway output can shift development from successful initiation to arrest or reduced growth. Together, these findings support the view that tooth agenesis frequently reflects dosage-sensitive network imbalance rather than single-gene determinism [35,36].

Collectively, these genetic data indicate that tooth agenesis does not typically arise from complete absence of developmental signals but from quantitative alterations in pathway strength, timing, or transcriptional responsiveness. The recurrent involvement of genes within converging signaling networks suggests that initiation failure reflects insufficient integrated signaling activity within specific odontogenic fields. Such a framework provides a mechanistic bridge between molecular genetics and spatial patterns of missing teeth, laying the foundation for a threshold-based interpretation of tooth initiation.

An additional consideration is the growing recognition that tooth agenesis often lies on a continuum between monogenic, oligogenic, and dosage-sensitive inheritance. Even within families carrying the same pathogenic variant, the number and distribution of missing teeth can differ substantially, implying that modifier loci, allelic dosage, or background variation alter the effective strength of the odontogenic network [10,11,34,35,36]. This is especially relevant for *WNT10A* and *EDA*-related phenotypes, in which heterozygous, compound heterozygous, or combined pathway perturbations may produce graded clinical presentations rather than discrete categories. Such observations strongly support a threshold-based interpretation in which cumulative signaling output, rather than mutation identity alone, determines whether a given tooth germ progresses beyond initiation.

Genome-wide evidence also supports a broader genetic architecture of tooth agenesis. Large-scale association studies have identified both rare and common variants contributing to overall and selective tooth agenesis, indicating that clinically similar phenotypes may arise from different combinations of high-impact variants and background genetic susceptibility [37]. This observation is consistent with a threshold model in which multiple genetic inputs can cumulatively shift odontogenic signaling output across critical developmental boundaries.

The same framework may also help reconcile the apparent boundary between syndromic and non-syndromic agenesis. Genes such as *EDA* influence multiple ectodermal appendages, yet their dental manifestations vary widely across individuals and may occur in isolation or as part of broader ectodermal phenotypes [34]. This suggests that tissue-specific thresholds for pathway activity differ across developmental systems. Teeth that initiate in more susceptible posterior fields may therefore fall below threshold before hair, glands, or other ectodermal structures exhibit overt dysfunction. A systems-level view of dosage sensitivity thus provides a biologically plausible explanation for why similar pathway disruptions can yield isolated hypodontia in one patient and more complex phenotypes in another.

Representative genes and pathway modulators supporting this interpretation are summarized in Table 1.

### Developmental Field Theory and Spatial Susceptibility

Developmental field concepts propose that teeth do not arise as completely independent units but as elements within spatially organized odontogenic regions. Within such fields, neighboring teeth share developmental signals, timing, and tissue competence, while individual tooth germs may differ in their sensitivity to perturbation. This helps explain why agenesis often affects recurring tooth classes rather than occurring randomly across the dentition.

In the present framework, developmental fields are interpreted as molecularly graded territories in which epithelial and mesenchymal tissues differ in signaling redundancy, competence, and threshold proximity. A tooth field operating close to the minimal level of required Wnt/BMP/FGF/SHH activity would be more vulnerable to modest reductions in pathway dosage, whereas a more robust field might remain above threshold despite the same genetic perturbation. This interpretation links classical field theory with molecular signaling and provides a bridge between clinical missing-tooth patterns and developmental mechanisms.

## 5. Threshold Model

The integration of developmental signaling networks and genetic modulation supports a threshold-based interpretation of tooth initiation. Within this framework, successful formation of each tooth germ may require that cumulative signaling activity within its specific odontogenic field exceeds a biological activation threshold. Tooth agenesis can therefore be interpreted as a failure of integrated pathway activity—rather than isolated gene dysfunction alone—to reach the level necessary for initiation.

Odontogenic signaling is both quantitative and spatially graded. Wnt, BMP, and FGF activity interact through feedback loops that stabilize epithelial–mesenchymal communication during the lamina-to-bud transition [3,5]. These interactions create a dynamic signaling landscape along the dental arch in which individual tooth germs are specified within overlapping developmental fields [2,12]. Posterior regions of the arch may operate closer to minimal signaling requirements, rendering them more sensitive to modest reductions in pathway intensity.

Under a threshold model, genetic variants affecting signaling amplitude reduce the effective strength or duration of pathway activation without necessarily abolishing it. When integrated activity remains above the activation threshold, tooth initiation would be expected to proceed, whereas signaling falling below this threshold within a given field may result in initiation failure and absence of the corresponding tooth germ. The number and distribution of missing teeth therefore depend on how far signaling output is shifted relative to field-specific thresholds (Figure 3). Although the activation threshold is conceptual in this framework, it may correspond biologically to minimal levels of β-catenin stabilization or mesenchymal transcriptional activation required for sustained tooth germ initiation. The threshold concept is also supported by inverse dental phenotypes associated with excessive rather than insufficient pathway activity. Whereas reduced Wnt/β-catenin signaling has been implicated in tooth agenesis and microdontia, overactivation of Wnt/β-catenin and SHH signaling has been associated with supernumerary tooth formation [38]. This bidirectional relationship strengthens the interpretation of tooth number as a quantitative output of pathway activity rather than a simple presence-or-absence effect of individual genes.

This interpretation accounts for several consistent clinical observations. First, missing-tooth patterns are not uniform across studies, but second premolars, maxillary lateral incisors, and third molars are repeatedly implicated depending on whether third molars are included and on the population studied [11]. These teeth likely arise in fields characterized by comparatively lower signaling redundancy, making them more vulnerable to quantitative reductions in pathway activity. Second, phenotypic variability among individuals carrying identical variants can be explained by differences in gene dosage, modifier loci, and temporal fluctuations in pathway activation. When signaling operates near threshold levels, minor variation in transcriptional responsiveness or pathway feedback may determine whether a specific tooth germ successfully initiates.

Arch asymmetry can also be interpreted within this model. Differences in spatial patterning, timing of initiation, or local signaling reinforcement between maxillary and mandibular fields may produce arch-specific vulnerability, even under similar genetic backgrounds. Such variation does not require distinct gene sets for each arch but may emerge from differential sensitivity to shared signaling gradients [12].

Importantly, the threshold model does not contradict established gene–phenotype associations. Rather, it reframes them within a systems-level perspective in which odontogenesis is governed by integrated pathway dynamics. Mutations in *PAX9*, *MSX1*, *WNT10A*, or *AXIN2* can be understood as modulators of signaling amplitude or stability that shift cumulative activity toward or away from field-specific activation thresholds [6,9,10,11]. The resulting phenotype reflects the degree of deviation relative to these thresholds rather than the identity of the mutated gene alone.

This model generates testable predictions. Individuals with mild hypodontia would be expected to show partial reduction in pathway activity rather than complete loss of signaling, whereas more severe oligodontia would be expected to reflect broader impairment of integrated signaling strength. In future studies, measurements such as β-catenin activity, transcriptional output, or mesenchymal responsiveness in appropriate developmental models could be used as indirect experimental correlates of the proposed threshold concept. Because such thresholds have not yet been quantified in human tissues, these predictions should be regarded as hypothesis-generating rather than established quantitative benchmarks.

By conceptualizing tooth initiation as a threshold-dependent process within spatially graded signaling fields, this framework provides a coherent explanation for phenotypic heterogeneity, distal tooth susceptibility, and variable penetrance in human tooth agenesis.

This threshold-based interpretation differs from previous conceptual models in two important ways. First, unlike directional or field-based frameworks that chiefly describe spatial patterns of tooth-number regulation, it explicitly links those patterns to quantitative pathway output within epithelial–mesenchymal signaling networks [18]. Second, unlike gene-centered reviews that catalogue causal variants and associated phenotypes, it seeks to explain why diverse variants converge on recurrent patterns of missing teeth by shifting a shared odontogenic system toward or away from critical activation boundaries [17]. The model therefore does not replace existing developmental or genetic classifications; rather, it provides a mechanistic layer that integrates them and generates experimentally testable predictions.

It is important to distinguish evidence-supported components of this framework from model-based extrapolations. The roles of Wnt, BMP, FGF, SHH, *EDA*, *MSX1*, *PAX9*, *WNT10A*, and *AXIN2* in odontogenesis or tooth agenesis are supported by experimental, genetic, or clinical evidence. By contrast, the precise numerical threshold at which individual human tooth fields fail to initiate has not yet been directly measured. Field-specific threshold positioning, arch asymmetry, and graded signaling failure should therefore be interpreted as testable hypotheses derived from the available evidence rather than as established quantitative parameters.

## 6. Implications

The proposed framework has implications for both biological interpretation and clinical reasoning. By viewing tooth agenesis as a quantitative failure of integrated signaling within spatially graded developmental fields, it shifts emphasis from isolated mutations to pathway-level dynamics and their modulation by epigenetic or environmental factors.

From a clinical standpoint, the pattern of missing teeth may provide indirect information about the degree and distribution of pathway insufficiency. Mild posterior hypodontia may reflect marginal reduction in signaling within more susceptible fields, whereas severe oligodontia may indicate broader or earlier impairment of integrated pathway activity. This perspective encourages clinicians to interpret symmetry, arch distribution, dentition type, and associated ectodermal features as biologically informative rather than purely descriptive findings.

The *AXIN2* example illustrates why this approach can matter beyond dentistry. Germline *AXIN2* variants have been linked to severe tooth agenesis and colorectal neoplasia risk [10,39]. Although this does not mean that all patients with hypodontia require gastrointestinal surveillance, severe or familial oligodontia—especially when accompanied by a family history of colorectal polyps or cancer—should prompt consideration of genetic counseling and appropriate medical referral.

From a translational perspective, the framework generates measurable questions for future research. Functional assays of pathway activity, organoid-based epithelial–mesenchymal models, and single-cell or spatial transcriptomic approaches may help relate genotype, pathway dosage, and phenotype more precisely. Standardized phenotyping of deciduous and permanent dentitions, including explicit treatment of third molars, will be especially important for testing whether clinically observed patterns truly reflect field-specific threshold behavior [10,17,34,39].

## 7. Limitations

This review has several limitations. First, the proposed threshold model is conceptual and is based on integration of published developmental, genetic, and clinical evidence rather than on new functional datasets. Second, much of the mechanistic evidence derives from animal and in vitro models, which may not fully recapitulate human dentition patterning. Third, genotype–phenotype correlations in tooth agenesis remain complicated by variable penetrance, oligogenic inheritance, and inconsistent phenotyping across studies. The framework presented here should therefore be viewed as a testable interpretative model rather than a definitive quantitative map of human odontogenesis.

A further limitation is the heterogeneity of clinical definitions and study designs across the literature. Reports differ in whether third molars are included, how deciduous versus permanent dentitions are analyzed, and whether isolated and syndromic cases are pooled or separated [17,34]. These differences can obscure field-specific patterns and weaken comparison between cohorts. In addition, most available human studies infer pathway effects from genotype and phenotype rather than directly measuring signaling amplitude in developing tissues. A major challenge for future work will therefore be to translate the conceptual threshold proposed here into experimentally quantifiable molecular parameters.

## 8. Future Directions

Future studies should integrate genomic sequencing, deep phenotyping, organoid or tooth germ models, and single-cell or spatial transcriptomics to identify field-specific signaling thresholds during tooth initiation. Such approaches may reveal how epithelial and mesenchymal subpopulations differ across susceptible tooth fields and how gene dosage or modifier loci shift pathway activity above or below initiation thresholds [40].

Equally important will be the development of genotype–phenotype datasets that capture not only which teeth are absent, but also symmetry, arch distribution, dentition type, and associated ectodermal features. Combining these structured phenotypes with functional readouts of Wnt, BMP, FGF, SHH, and *EDA* pathway activity may allow investigators to distinguish variants that primarily alter network amplitude from those that change timing, feedback stability, or spatial patterning. Ultimately, a quantitative framework of this kind may improve risk prediction, genetic counseling, and the design of regenerative or precision-dentistry strategies for patients with hypodontia and oligodontia [17,34,40].

## 9. Conclusions

Human tooth agenesis may be most usefully interpreted as a quantitative developmental disorder in which integrated signaling activity fails to exceed field-specific initiation thresholds within odontogenic tissues. In this view, variants in genes such as *MSX1*, *PAX9*, *WNT10A*, *AXIN2*, and *EDA* alter pathway amplitude, timing, or responsiveness rather than acting as simple binary determinants of tooth presence. The signaling-threshold framework proposed here integrates molecular genetics with developmental field theory and offers a testable explanation for distal tooth susceptibility, variable penetrance, and heterogeneous agenesis patterns in humans.

## Figures and Tables

**Table 1:** Representative genes and pathway modulators implicated in tooth agenesis and their interpretation within a signaling-threshold framework.

Gene/Modulator	Pathway or Molecular Axis	Evidence/Typical Phenotype	Interpretation Within the Threshold Framework
***PAX9*** [9,29]	BMP-associated mesenchymal competence; supports BMP-mediated transcriptional reinforcement and bud stabilization.	Posterior oligodontia, especially molars and premolars.	Reduced mesenchymal competence may shift susceptible posterior fields below the initiation threshold.
***MSX1*** [5,6,30]	BMP downstream transcriptional mediator; supports mesenchymal responsiveness and feedback stability.	Hypodontia involving premolars and third molars.	Weakened epithelial–mesenchymal reinforcement may impair the signaling needed to sustain initiation.
***WNT10A*** [11]	Canonical Wnt ligand; contributes to β-catenin-dependent transcription.	Broad spectrum from mild hypodontia to oligodontia, often involving second premolars and third molars.	Represents a dosage-sensitive phenotype in which severity may reflect the degree of pathway reduction.
***AXIN2*** [10]	Negative regulator of Wnt signaling; affects β-catenin turnover and pathway amplitude.	Variable hypodontia with familial heterogeneity.	Shows that altered network dynamics, not only loss of signal, can determine the clinical phenotype.
***EDA*** [34]	*EDA–EDAR–EDARADD*/NF-κB axis; modifies ectodermal tissue responsiveness and tooth number regulation.	Deciduous tooth agenesis and ectodermal-spectrum phenotypes.	Supports tissue-specific threshold sensitivity and helps explain the continuum between isolated and syndromic agenesis.
***KCTD1*** [36]	WNT–SHH–BMP crosstalk; affects β-catenin levels and cross-pathway balance.	Isolated dental anomalies.	Small shifts in pathway crosstalk may be sufficient to alter initiation outcome in susceptible fields.
***FGF8* dysregulation** [27,35]	FGF positional and proliferative signaling; influences field boundaries and mesenchymal support.	Incisor agenesis and molar microdontia.	Quantitative spatial signaling changes may affect both initiation probability and subsequent patterning.
***BMPR2*** [32]	BMP receptor-level signaling and mesenchymal responsiveness.	Reported in nonsyndromic oligodontia.	Supports the view that impaired BMP responsiveness at receptor level can reduce odontogenic competence below the initiation threshold in susceptible fields.

Note: The table is intentionally selective and includes representative genes and pathway modulators that illustrate different levels of threshold regulation, including ligands, receptors, transcription factors, and pathway feedback regulators.

## Data Availability

No new data were created or analyzed in this study. Data sharing is not applicable to this article.
